# Dehydration-Responsive Left-Handed G-Quadruplexes Expand the Sequence Repertoire

**DOI:** 10.64898/2026.10.05.756892

**Published:** 2026-10-13

**Authors:** Yun Chen, Andrew Hendrickson, Julien Gros, Eric Xing, Eleano R. Windle, Jiawei Wang, Robert M. Edkins, Susan J. Quinn, Liliya A. Yatsunyk, Jean-Louis Mergny

## Abstract

G-quadruplexes (G4s) are widely studied noncanonical nucleic-acid structures that carry important biological functions. Typically, G4s form from G-rich sequences containing at least four tracts of more than two consecutive guanines, yet a plethora of unconventional G4-forming sequences have been identified, including sequences that fold into G4s only under specific conditions. These G4 states remain poorly resolved. Here we identify dehydration-responsive left-handed G4s (drLHG4s), a class of left-handed G4 (LHG4) structures accessible under dehydrating conditions. Using d(GT)n repeats, we show that left-handed folding depends on repeat length, salt condition and solvent conditions. Systematic mutational analysis reveals a broader but register-constrained sequence space, while high-resolution crystal structures of d-GTGGTGTGTGGTG, a sequence that folded robustly under dehydrating conditions, establish a bulged LHG4 architecture and reveal a distinctive hydration sphere. We further show that selected peptides and a cationic zinc phthalocyanine derivative, ZnTEGQPyrPc, containing four triethylene glycol methyl ether tails can induce LHG4 states. Together, our findings reveal a dehydration-responsive G4 landscape and expand the experimentally accessible sequence space of LHG4 folding.

